# Reconstruction of gross avian genome structure, organization and evolution suggests that the chicken lineage most closely resembles the dinosaur avian ancestor

**DOI:** 10.1186/1471-2164-15-1060

**Published:** 2014-12-11

**Authors:** Michael N Romanov, Marta Farré, Pamela E Lithgow, Katie E Fowler, Benjamin M Skinner, Rebecca O’Connor, Gothami Fonseka, Niclas Backström, Yoichi Matsuda, Chizuko Nishida, Peter Houde, Erich D Jarvis, Hans Ellegren, David W Burt, Denis M Larkin, Darren K Griffin

**Affiliations:** School of Biosciences, University of Kent, Canterbury, CT2 7NJ UK; Department of Comparative Biomedical Sciences, Royal Veterinary College, University of London, London, NW1 0TU UK; Department of Pathology, University of Cambridge, Tennis Court Road, Cambridge, CB2 1QP UK; Department of Evolutionary Biology, Evolutionary Biology Centre, Uppsala University, Norbyvägen 18D, SE-752 36 Uppsala, Sweden; Laboratory of Animal Genetics, Department of Applied Molecular Biosciences, Graduate School of Bioagricultural Sciences, Nagoya University, Furo-cho, Chikusa-ku, Nagoya, Aichi 464-8601 Japan; Department of Natural History Sciences, Faculty of Science, Hokkaido University, Kita 10, Nishi 8, Kita-ku, Sapporo, Hokkaido 060-0810 Japan; Department of Biology, New Mexico State University, Las Cruces, NM 88003 USA; Department of Neurobiology, Duke University Medical Center, Box 3209, Durham, NC 27710 USA; Department of Genomics and Genetics, The Roslin Institute and Royal (Dick) School of Veterinary Studies, University of Edinburgh, Edinburgh, EH25 9PS UK; School of Human and Life Sciences, Canterbury Christ Church University, Canterbury, Kent CT1 1QU UK

**Keywords:** Ancestral karyotype, Avian genome, Chromosome evolution, Dinosaur

## Abstract

**Background:**

The availability of multiple avian genome sequence assemblies greatly improves our ability to define overall genome organization and reconstruct evolutionary changes. In birds, this has previously been impeded by a near intractable karyotype and relied almost exclusively on comparative molecular cytogenetics of only the largest chromosomes. Here, novel whole genome sequence information from 21 avian genome sequences (most newly assembled) made available on an interactive browser (Evolution Highway) was analyzed.

**Results:**

Focusing on the six best-assembled genomes allowed us to assemble a putative karyotype of the dinosaur ancestor for each chromosome. Reconstructing evolutionary events that led to each species’ genome organization, we determined that the fastest rate of change occurred in the zebra finch and budgerigar, consistent with rapid speciation events in the Passeriformes and Psittaciformes. Intra- and interchromosomal changes were explained most parsimoniously by a series of inversions and translocations respectively, with breakpoint reuse being commonplace. Analyzing chicken and zebra finch, we found little evidence to support the hypothesis of an association of evolutionary breakpoint regions with recombination hotspots but some evidence to support the hypothesis that microchromosomes largely represent conserved blocks of synteny in the majority of the 21 species analyzed. All but one species showed the expected number of microchromosomal rearrangements predicted by the haploid chromosome count. Ostrich, however, appeared to retain an overall karyotype structure of 2*n* = 80 despite undergoing a large number (26) of hitherto un-described interchromosomal changes.

**Conclusions:**

Results suggest that mechanisms exist to preserve a static overall avian karyotype/genomic structure, including the microchromosomes, with widespread interchromosomal change occurring rarely (e.g., in ostrich and budgerigar lineages). Of the species analyzed, the chicken lineage appeared to have undergone the fewest changes compared to the dinosaur ancestor.

**Electronic supplementary material:**

The online version of this article (doi:10.1186/1471-2164-15-1060) contains supplementary material, which is available to authorized users.

## Background

The mechanisms of genome evolution are most often considered from the perspective of individual genes or gene families; there is nonetheless increasing evidence supporting the functional role and significance of events at a chromosomal (cytogenetic) level [1]. To date, bird genomes remain relatively understudied from an overall genome organization perspective; however, the recent availability of multiple avian genome sequence assemblies [2] allows us to consider the role of chromosomal change in the evolution of Aves from their dinosaur ancestors. Chromosome rearrangements between species can cause or reinforce reproductive isolation through reduced fitness of hybrid offspring due to a compromised ability to synapse and segregate chromosomes at meiosis [3, 4]. Moreover, reduced interspecific recombination in rearranged regions is thought to promote the accumulation of incompatibility loci in such regions [5–7]. The purpose of this study was to gain further insight into the mechanism of bird evolution through the multiple comparative analyses of chromosomal segments and breakpoints.

Unraveling the mechanisms and relevance of bird karyotype evolution has hitherto been impeded by a karyotype that is difficult to define because of indistinct banding on the macrochromosomes and a preponderance of cytogenetically indistinguishable microchromosomes. Indeed, to date, only a single avian karyotype (chicken) has been fully defined using a combination of BAC/cosmid clones and chromosome paints generated by flow cytometry and microdissection [8]. Moreover, karyotypes are broadly similar in overall pattern from species to species. For instance, at a cytogenetic level, two thirds of bird species have a chromosome number of around 2*n* = 80 with similar numbers of macro- and microchromosomes suggesting little interchromosomal changes between species [9]. Molecular insights into interchromosomal differences between species (and the evolutionary events that have led to them) have focused mostly on the largest macrochromosomes. These studies applied chicken chromosome paints [10] to the chromosomes of numerous other species (reviewed in [11]) in zoo-FISH experiments. Such investigations have provided much insight into inter-macrochromosomal rearrangements between birds with the underlying message that the ancestral pattern has remained largely unaltered in the majority of species. Rare exceptions include significant chromosome rearrangement in Psittaciformes (parrots etc.), Falconiformes (falcons) and Sphenisciformes (penguins) [11]. There are also individual changes associated with representative orders, e.g., fission of chromosome 1 in Passeriformes (songbirds) and of chromosome 2 in certain Galliformes (land fowl) (reviewed in [11]). Studies of interchromosomal changes involving the microchromosomes are much more limited as the flow cytometry methods used to generate the chromosome paints [10] do not have the resolution to isolate individual microchromosomes.

Using chicken BAC clones, studies provide a low-resolution appraisal of intrachromosomal rearrangements between chicken and other species [12–14] (turkey, duck, zebra finch, respectively). This approach, however, is limited in its ability to identify the molecular coordinates of evolutionary breakpoints. The availability of whole assembled genomes [15–17] allows comparative genomics at a much more detailed level of resolution than can be achieved by cross-species FISH. Burt *et al.*[18] were the first to use bioinformatics to define inter-species analysis of whole avian chromosomes at a genomic level (chicken-human). The publication of the chicken genome sequence [15] provided more detailed information, establishing conserved synteny between chicken and human whole genome assemblies. In the ten years since, only conserved synteny comparisons have been made between the chromosomes of two [14, 19], or at most three [20, 21] avian species.

The use of whole genome assemblies to study cytogenetic phenomena has raised interest in the study of comparative cytogenetics from the perspective of evolutionary breakpoint regions (EBRs) and homologous synteny blocks (HSBs). To date, the majority of such studies have focused on mammals [22], however, analysis of other groups, such as birds, is essential in order to establish whether mammalian systems are representative of, or an exception to, general patterns observed in other animal groups. Larkin *et al.*[22] found that, in mammals, EBRs can lie in gene-dense regions. In the human genome EBRs also lie in regions with more zinc finger protein genes, more genes whose function is associated with environmental stimulus response, as well as more segmental duplications, CNVs, SNPs and retrotransposed genes. Such “EBR genes” appear to be related to lineage-specific biology and adaptive features [22–24]. EBRs are also frequently reused, i.e. there are regions of the genome that are prone to chromosomal breakage leading to translocations, inversions and fissions [25, 26]. Comparison of sequence assemblies in chicken, zebra finch and turkey suggests that breakpoint reuse is higher in birds than in mammals [20, 21]. The data in birds also suggests a key role for recombination-based mechanisms in the generation of chromosome rearrangements in that EBR location is consistent with elevated levels of genetic recombination at these loci [14]. This is consistent with the notion that, if recombination drives chromosomal rearrangements and assuming an evolutionarily conserved recombination landscape [27–29], EBRs might be enriched in genomic regions with elevated recombination rates. Not all species show an association of chromosomal breakage and elevated recombination however, e.g., insects [30, 31] and mammals. Indeed, in mammals Larkin *et al.*[22] suggested that the highest levels of recombination are located between the EBRs rather than in association with them.

HSBs have been defined in all animal species thus far examined for conserved chromosomal synteny [32]. Larkin *et al.*[22] argue that the continued presence of HSBs in all species may indicate a selective advantage to the retention of gene combinations in close proximity. Supporting evidence is found in the fact that multispecies HSBs (msHSBs) involving nine mammals plus chicken, unlike EBRs, are enriched in gene ontology (GO) terms for organismal development, central nervous system, and brain function in the human genome. Others argue that the idea of close proximity and any resulting correlation in expression patterns (if present) are not necessarily adaptive or required (e.g., [33, 34]). Given that around three quarters of avian chromosomes are small, cytogenetically indistinguishable microchromosomes, and that overall karyotype structure appears broadly similar between at least two thirds of bird species, a high degree of conserved chromosomal synteny is inferred [9]. This raises the hypothesis that avian karyotypes are evolutionarily static; however, for this to be tested, we would first need to establish that inter-microchromosomal rearrangements are rare or absent in most birds. If true, we would subsequently hypothesize that, like HSBs in mammals, individual whole microchromosomes are enriched for functional GO terms (regardless of any intrachromosomal rearrangements between them).

A detailed account of the chromosomal differences and changes that have occurred during the evolution of avian chromosomes is an essential prerequisite for any further insights into functional and/or mechanistic relevance. The combination of comparative analysis by bioinformatics and chromosome painting has the potential to do this, provided the appropriate tools are developed and used. The purpose of this study was thus to examine multiple avian genomes recently sequenced [2, 35], reconstruct the common ancestral karyotype and thence the evolutionary events that led to extant karyotypes. Furthermore, we tested the hypothesis that EBRs occurring in two lineages (chicken and zebra finch) are associated with elevated levels of genetic recombination and assessed the degree to which EBRs are reused in avian evolution. Finally, we tested the hypothesis that whole microchromosomes essentially constitute interchromosomal HSBs (i.e. that rearrangements between them are rare or absent) and that each microchromosome consists of functionally enriched GO terms.

## Results

### Genomic data and visualization of HSBs and EBRs

**Figure 1:**
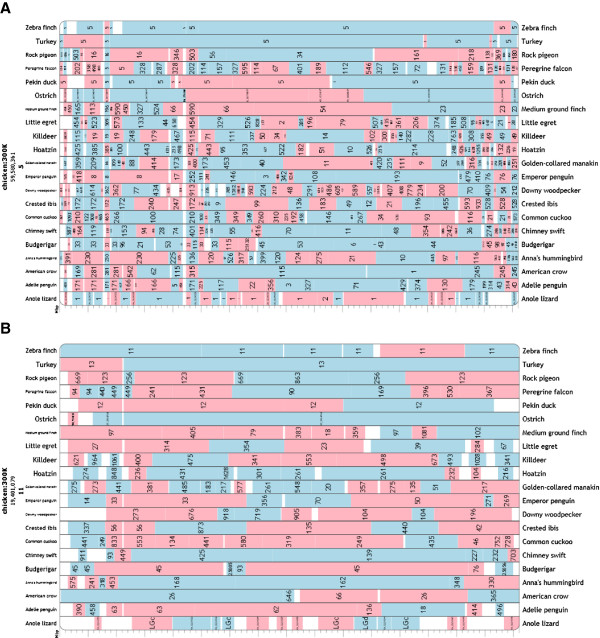
**Screenshots of Evolution Highway comparing 20 avian genomes plus Carolina anole lizard.** Shown relative to chicken chromosomes 5 **(A)** and 11 **(B)**. For turkey, zebra finch, duck and Carolina anole, numbers refer directly to chromosome assignment. For the remainder, numbers refer to scaffold assignments. Red segments are inversions.

Results from this study were derived from HSB and EBR data from a total of 21 avian genomes and one outgroup reptile species loaded to an interactive, publicly available chromosome browser Evolution Highway [36]. This now allows for multispecies cytogenetic comparison in birds [37]. For six bird species (chicken, turkey, Pekin duck, zebra finch and budgerigar) and one lizard outgroup (Carolina anole - *Anolis carolinensis*), a combination of large scaffold size (manifested by N50 > 10 Mb) and supporting molecular cytogenetic data (cross-species chromosome painting) allowed us to make chromosomal or near chromosomal comparison, orientation of HSBs and reconstruction of ancestral chromosome rearrangements. Evolution Highway screenshots for avian species and lizard outgroup compared to chicken chromosomes 5 and 11 are illustrated in Figure 1 (these chromosomes chosen throughout as they give the clearest representative examples in both FISH and bioinformatics analyses).

### FISH analysis

Reconstructions of scaffold-based assemblies also relied, in part, on previously published zoo-FISH (BAC and chromosome painting) data for the macro- and microchromosomes of chicken, turkey, duck and zebra finch [12–14] as well as newly generated data in this study as follows: we used seven new chicken microchromosomal paints A–G [21], verifying their assignments with chicken BACs (see Additional file 1) by dual color FISH and painting them onto ostrich and budgerigar metaphases.

**Figure 2:**
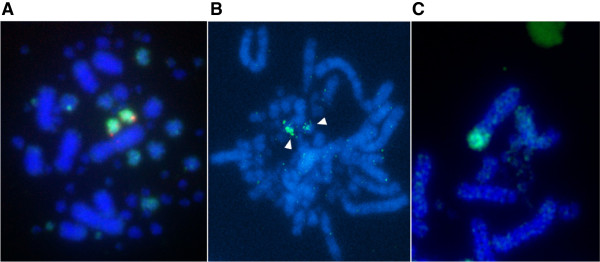
**Chromosome painting experiment using chromosome paint A. (A)** On chicken chromosomes; dual FISH with a chromosome 11 BAC (red) confirms that this chromosome paint (green) maps to chromosome 11. **(B)** Painting one chromosome pair in ostrich; and **(C)** painting the terminal *q* arm of chromosome 5 in budgerigar.

For chicken, turkey, duck and zebra finch, zoo-FISH has been previously described [12–14]. For ostrich, no further differences between this species and chicken microchromosomes were found (Table 1 and Figure 2). For budgerigar, analysis reveals a more complex pattern incorporating several of the microchromosomes, namely six hitherto undescribed fusions (Table 1 and Figure 2).

**Table 1:** **Comparative mapping of chicken chromosome paints A–G, and their ostrich and budgerigar orthologs**

Chromosome paint ID	Chicken chromosome(s)	Ostrich orthologs (all microchromosomes)	Budgerigar orthologs
A	11	1 pair	Fusion as part of chromosome 5
B	10 and 12	2 pairs	2 pairs of microchromosomes (no apparent fissions/fusions at this resolution)
C	13	1 pair	1 pair of microchromosomes (no apparent fissions/fusions at this resolution)
D	13 and 14	1 pair	1 microchromosome pair +1 arm of chromosome 8 = fission and fusion at this resolution
E	10 and 12	2 pairs	1 pair = fusion
F	16, 17 and 18	3 pairs	2 pairs = fusion
G	~5 pairs smaller than 18	No result	3 pairs = 2 fusions (although some signals are weak so may be failure of hybridization)

Note:

Bioinformatic approaches detected further rearrangements that are beyond the resolution of zoo-FISH.

BACs that confirmed these assignments are given in Additional file 1: Table S1.

### Reconstruction of ancestral karyotypes and chromosomal changes

**Figure 3:**
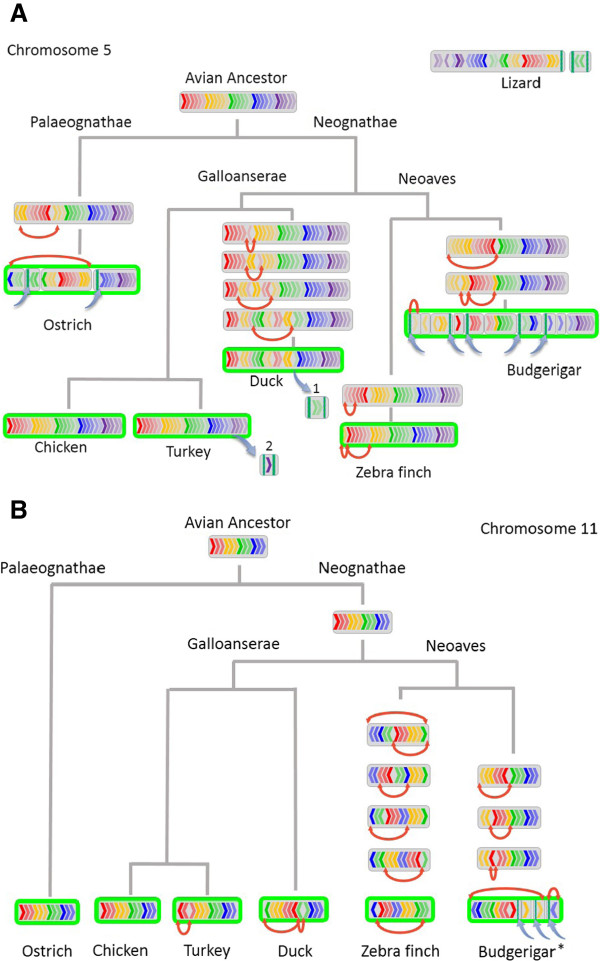
**Ancestral arrangement of chromosomes in six species and the rearrangements led to the extant pattern.** Exemplified for chicken chromosomes 5 (**A**; Carolina anole lizard arrangement also indicated) and 11 **(B)**. Rainbow patterned arrows within the chromosomes represent the HSBs, red curved arrows indicate chromosome inversions, blue arrows indicate chromosome translocations, green outline indicates the chromosome painting results. As the arrangement for ostrich and Neognathae ancestors were the same, the avian ancestor could be derived (unlike for other chromosomes smaller than 5). *In budgerigar, FISH indicates fusion to a larger chromosome.

A combination of FISH and bioinformatic analyses allowed reconstruction of ancestral chromosomes 1–5 for all birds, and chromosomes 6–28 + Z for Neognathae (see Methods). As a frame of reference, we used the new phylogenetic tree of another recent study [35]. Figure 3A indicates the comparative genomics of ancestral chromosome 5 and its orthologs, and 3B the changes that occurred in the orthologs of chicken chromosome 11. Although the outgroup did not have sufficient coverage to generate an “all-avian” ancestral chromosome directly for chromosome 11, the avian ancestral rearrangement is inferred from the identical patterns present in ostrich and chicken.

**Figure 4:**
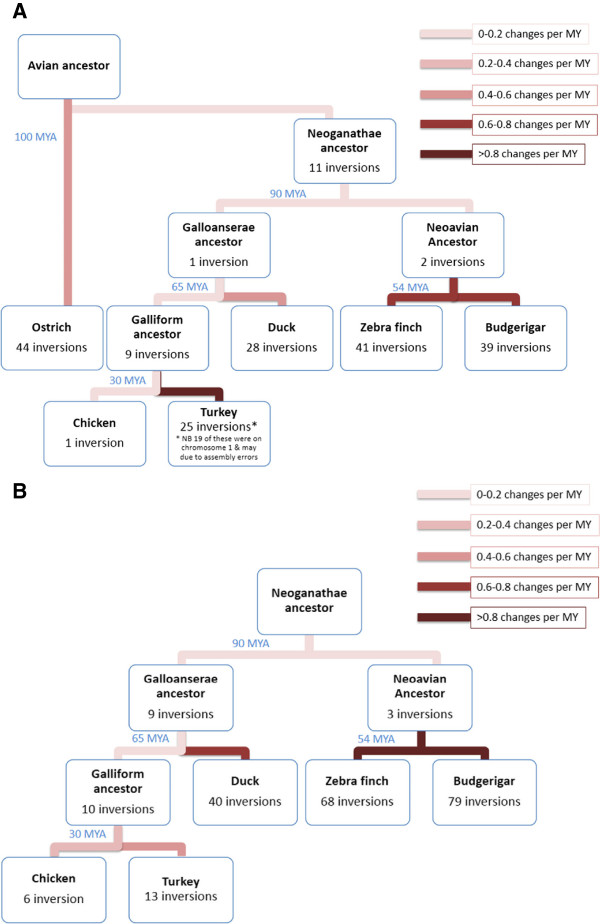
**Total number of chromosomal inversions in six extant species as they diverged from the ancestor.** The inversions most parsimoniously explain the patterns seen in these species. **(A)** For chromosomes 1–5, sufficient coverage of the lizard outgroup allowed conclusions to be drawn from an avian ancestor. **(B)** For chromosomes 6–28 + Z, ostrich was used as an outgroup due to the lack of coverage in the lizard. Greatest rates of change were seen in zebra finch and budgerigar. The phylogenetic tree is based on [35].

Overall, analysis suggests that, of the six species, the chicken lineage underwent the least number of intrachromosomal rearrangements (i.e. chicken was most similar to the common avian ancestor, probably a bipedal feathered dinosaur). Of the 46 rearrangements observed in the turkey lineage since the divergence from chicken 30 MYA (million years ago), 19 were on chromosome 1 (we believe that this may be a slight overestimate due to assembly errors in the turkey genome). The analysis also suggests that ostrich lineage underwent 44 intrachromosomal changes on chromosomes 1–5 since the divergence from the common avian ancestor (approximately 100 MYA), and the duck 28 changes since the galliform-anseriform divergence (~65 MYA). A faster rate of change was seen in the zebra finch and the budgerigar lineages, 41 in the former and 39 in the latter, occurring since the passeriform-psittaciform divergence (~54 MYA, Figure 4A). For the orthologs of chromosomes 6–28 + Z, in the absence of meaningful data from the lizard outgroup (i.e. there was minimal comparative data available), our analysis focused on the Neognathae alone (using ostrich as an outgroup, Figure 4B). Again the chicken lineage appeared to have the least number of changes compared to the ancestor and the greatest rate of change was seen in the zebra finch since the passeriform-psittaciform divergence 54 MYA (68 for zebra finch and 79 for budgerigar). For all chromosomes, the intrachromosomal events are most parsimoniously explained by a series of inversions, and the interchromosomal rearrangements by a series of translocations. We next tested the robustness of our analysis in a series of additional MGRA simulations and iterations, excluding one species at a time from the set of six species (see Methods). We were interested to know if this would affect the general chicken-like pattern of the reconstructed avian ancestor. Results showed that, although the number of reconstructed contiguous ancestral regions (CARs) tended to decrease slightly if more fragmented (scaffold-based) genome assemblies (i.e. those of budgerigar and ostrich) were excluded, near identical order of msHSBs were observed within each CAR regardless of excluding one species. The number of changes and their timescales (hence rates of change) are presented in Figure 4A (for all avian chromosomes 1–5) and 4B for the Neognathae (chromosomes 6–28 + Z).

A combination of FISH and bioinformatic data revealed a total of 26 interchromosomal and 44 intrachromosomal changes that have occurred in the ostrich lineage since divergence of the common avian ancestor ~100 MYA (Table 2 and Figure 4A). Most changes that occurred in the duck, chicken and turkey lineages appear to have done so since the galliform-anseriform divergence ~65 MYA. Notably, most of the changes seen in budgerigar and zebra finch lineages each appear to be different from one another, thereby suggesting that nearly all changes have occurred in the ~54 million years since the Passeriformes and the Psittaciformes diverged (Figure 4 and Table 2).

**Table 2:** **Total numbers of inter- and intrachromosomal rearrangements since divergence from avian ancestor 100 MYA**

Species	Ostrich	Chicken	Turkey	Duck	Zebra finch	Budgerigar
No. of interchromosomal changes (as determined by FISH) from avian ancestor	0	1	1	0	2	8
No. of interchromosomal changes (determined using bioinformatics) from avian ancestor	26	1	5	1	2	40
No. of intrachromosomal changes from avian ancestor in chromosomes 1–5 (excluding 4p)	44	22	46	40	54	52
No. of intrachromosomal changes from Neognathae ancestor in chromosomes 6–28 + 4p + Z	Not applicable	25	32	49	71	82

Closer analysis of the breakpoints to address the question of breakpoint reuse (see Background) identified, in chicken chromosomes 1–5 (and their turkey, duck, zebra finch, budgerigar and ostrich orthologs), 620 segment ends, of which 421 were involved in rearrangements. The most parsimonious predicted pathways from the common avian ancestor suggested that 100 breakpoint regions (23.8%) recurred in different lineages, whereas 214 breakpoint regions (50.8%) recurred in either the same or different lineages. In chicken chromosomes 4p, 6–28 and Z, and their turkey, duck, zebra finch and budgerigar orthologs, 560 segment ends were identified, of which 428 were involved in rearrangements. The most parsimonious predicted pathways from the common avian ancestor suggested that 109 breakpoint regions (25.5%) recurred in different lineages, whereas 210 breakpoint regions (49.1%) recurred in either the same or different lineages.

### EBRs and recombination in chicken and zebra finch

As also mentioned in the Background section, we tested the hypothesis that the presence of EBRs was related to the regional recombination rate. Given the quality of the genetic maps and the data available in this study, this could be achieved for the chicken and zebra finch only.

**Figure 5:**
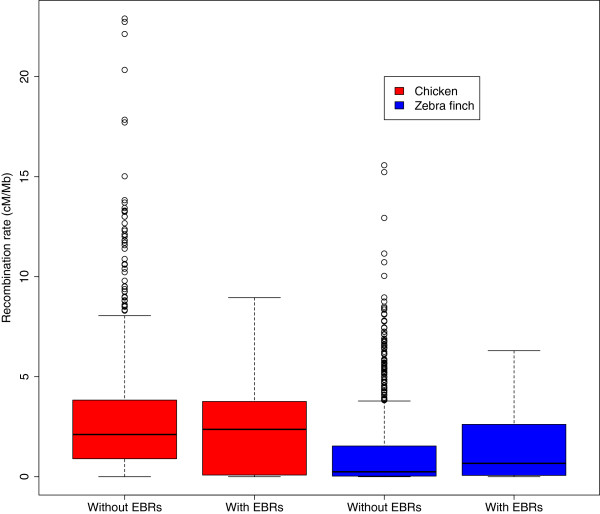
**Rates of recombination and their association with EBRs for chicken (red) and zebra finch (blue).** In chicken, recombination rates are near identical in windows with and without EBRs (2.90 and 2.80, respectively). In zebra finch recombination rates are slightly higher in windows with EBRs (1.60 and 1.29, respectively) but the difference does not reach statistical significance (*P* = 0.1 for both tests used).

In chicken the analysis revealed no association between presence of EBR and the regional recombination rate. The 1 Mb non-overlapping windows containing EBRs (*n* = 35) had an average recombination rate of 2.80 (±3.00, SD) cM/Mb while windows without EBRs (*n* = 963) had an average recombination rate of 2.90 (±3.00) cM/Mb (Wilcoxon’s test, *W* = 13492, *P* = 0.42; randomization test, empirical difference in mean between classes = -0.11, *P* = 0.28; Figure 5).

In zebra finch, 1 Mb non-overlapping windows with EBRs (*n* = 31) had a slightly higher recombination rate than windows without (*n* = 952; 1.60 vs. 1.29 cM/Mb), although this was not statistically significant (Wilcoxon’s test, *P* = 0.1; randomization test, empirical difference in mean between classes = 0.31, *P* = 0.1; Figure 5).

### Interchromosomal changes in multiple species and GO of microchromosomes

**Figure 6:**
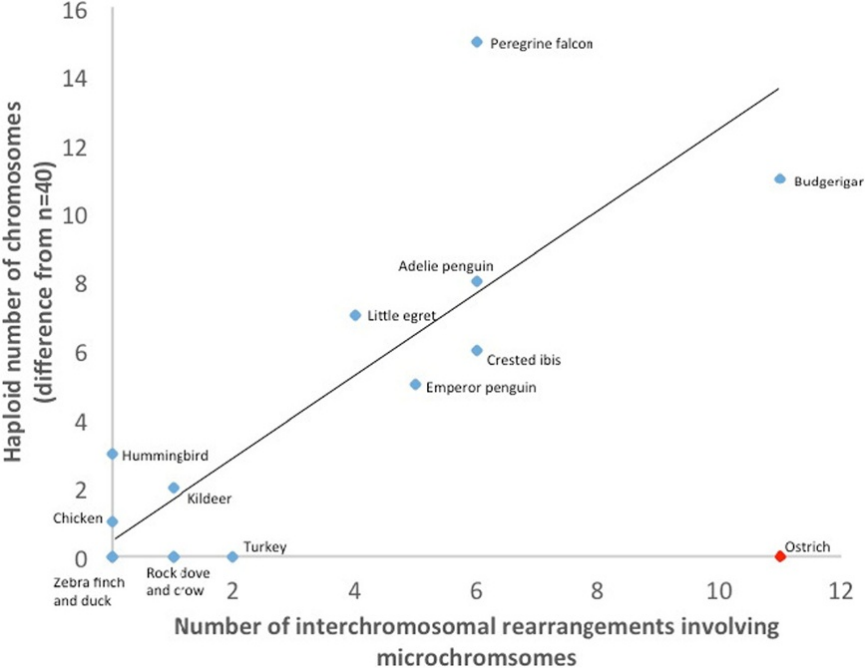
**Number of interchromosomal rearrangements involving microchromosomes.** Plotted against deviation from *n* = 40 for each species in which chromosome number is published (Table 3). Analysis suggests that haploid chromosome number effectively is a reflection of number of microchromosomal rearrangement, except in ostrich (red dot). Best-fit line is drawn excluding ostrich outlier (*R* ^*2*^ = 0.7, *P* = 0.0002 if ostrich is excluded; *R* ^*2*^ = 0.3, *P* = 0.03 if ostrich is included).

For chicken, turkey, zebra finch and duck, inter-macrochromosomal changes have been previously described, i.e. chromosome 4 fusion for chicken, chromosome 2 fission for turkey, chromosome 1 fission for zebra finch, and no changes in duck [12–14] in these four species. In the current analyses, however, results suggested that there were at least 26 interchromosomal differences between chicken and ostrich, and 40 between chicken and budgerigar for all chromosomes (Table 2), with the changes in the budgerigar lineage occurring since the passeriform-psittaciform divergence (~54 MYA). Considering microchromosomes alone and using data pertaining to numbers of interchromosomal rearrangements for the remaining 15 species [37], results suggested that microchromosomal rearrangement was rare, except where the species of interest had been previously known to have an unusually large or small number of chromosomes (Table 3). In other words, as illustrated in Figure 6, there was a statistically significant correlation (*R*^*2*^ = 0.3; *P* = 0.03) between number of interchromosomal rearrangements and published deviation from a haploid chromosome number of 40. The exception to this “rule” was the ostrich (2*n* = 80), with 26 interchromosomal differences, 11 involving the microchromosomes, results suggesting significant rearrangement while maintaining the overall karyotypic structure. Indeed, if ostrich is excluded from the analysis outlined in Table 3 and Figure 6, the statistical significance of the association increases markedly (*R*^*2*^ = 0.7, *P* = 0.0002).

**Table 3:** **Total number of interchromosomal rearrangements involving microchromosomes in 21 avian species compared to chicken**

Species	Total number of interchromosomal changes involving macro- and microchromosomes	Interchromosomal changes between microchromosomes only	Haploid chromosome number (difference from***n*** = 40)
Adélie penguin (*Pygoscelis adeliae*)	6	0	48 (8)
American crow (*Corvus brachyrhynchos*)	1	0	40 (0)
Common cuckoo (*Cuculus canorus*)	1	0	?
Pekin duck (*Anas platyrhynchos*)	0	0	40 (0)
Little egret (*Egretta garzetta*)	4	1	33 (7)
Emperor penguin (*Aptenodytes forsteri*)	5	0	36 (5)
Peregrine falcon (*Falco peregrinus*)	6	4	25 (15)
Zebra finch (*Taeniopygia guttata*)	0	0	40 (0)
Hoatzin (*Ophisthocomus hoazin*)	3	0	?
Anna’s hummingbird (*Calypte anna*)	0	0	37 (3)
Crested ibis (*Nipponia nippon*)	6	0	34 (6)
Killdeer (*Charadrius vociferous*)	1	0	38 (2)
Golden collared manakin (*Manacus vitellinus*)	0	0	?
Medium ground finch (*Geospiza fortis*)	0	0	?
Ostrich (*Struthio camelus*)	11	0	40 (0)
Budgerigar (*Melopsittacus undulates*)	11	2	29 (11)
Rock dove (*Columba livia*)	1	0	40 (0)
Chimney swift (*Chaetura pelagica*)	1	0	?
Turkey (*Meleagris gallopavo*)	2	0	40 (0)
Downy woodpecker (*Picoides pubescens*)	4	1	?
Chicken (*Gallus gallus*)	0	0	39 (1)

As detected by bioinformatic approaches [37] and compared to the published haploid number of chromosomes in each species [9]. For counts of all interchromosomal rearrangements in the bird genomes see [37].

Once we had established (above) that rearrangements were rare in the microchromosomes, then this led to the hypothesis that each microchromosome contained functionally enriched GO categories (see Background). We found evidence to support this hypothesis only for chromosome 16 (enriched for immune function) when *P* < 0.05 and a false discovery rate (FDR) threshold of 0.05 were applied. Nonetheless several chromosomes had a significant *P* value but did not pass the FDR threshold: for chromosome 11 enrichment categories were apparent for drug/caffeine metabolism as well as hemophilic cell adhesion; for chromosome 12 genes for nucleotide binding were clustered together; for chromosome 13 there were enrichment categories for GTPase regulator activity; phosphatase activity in chromosome 15; chromosome 17 for glycosylation and glycoprotein related processes; chromosome 18 for cytoskeletal and motor protein related genes; and chromosome 20 for genes involved in apoptosis and cell death.

We thus find evidence to support our hypothesis that microchromosomes represent highly conserved blocks of interchromosomal synteny but find limited evidence to support the hypothesis that one possible explanation for this is a clustering of genes of associated function on the same chromosome.

## Discussion

The results presented here signify the most comprehensive appraisal of avian comparative cytogenetics to date. They provide a more detailed reconstruction of avian genome evolution than could be achieved by zoo-FISH analysis alone and demonstrate proof of principle from which further studies of genome evolution and comparative genomics can ensue.

We used a highly interactive avian genome dataset from the Evolution Highway comparative chromosome browser [37, 38] that, as has already been demonstrated in mammals, can be applied to compare the chromosome organization of individual or multiple species. The ultimate aim for this browser is that, in chromosomes for all avian species uploaded, HSBs will be displayed with reference to the chromosome number, as is currently the case for turkey, zebra finch and duck, or to specific scaffolds for other birds. In future, this will be achieved by a number of strategies: (a) by improved scaffold sizes, e.g., using optical mapping such as has been achieved to some degree in ostrich and budgerigar in this study; (b) by linkage to radiation hybrid (RH) maps such as was achieved for duck in this study (see also [19]); (c) by association with known linkage and other physical maps (e.g., [39, 40]); d) by use of novel algorithms to order and orient scaffolds into longer chromosomal fragments or whole chromosomes using comparative genome information and pair-end reads (reference-assisted chromosome assembly; [41]); (e) by systematic FISH mapping to chromosomes of orthologous clones derived from the individual scaffolds. We are currently concentrating our efforts on the development of FISH probes that will identify not only on which chromosomes the scaffolds lie in the species of interest, but also the order in which they appear on the chromosome. With current technology, however, even the best-assembled genomes (e.g., assisted with optical mapping) require a degree of intervention by molecular cytogenetics in order to generate a complete picture of overall genome organization. Given the efforts that have been made to sequence the genomes of the birds recently by current technologies [2], it is questionable how many of them will be re-sequenced using newer technologies that generate large scaffolds. A note of caution is relevant here: no genome assembly is “perfect” - the results reported here and elsewhere represent the state of the art in terms of what can be reasonably gleaned with the current technology available. Our future studies will focus on the systematic molecular characterization by zoo-FISH of as many scaffolds and EBRs as time and resources allow.

Earlier cytogenetic data suggested that, for the majority of bird species, karyotypic patterns are broadly similar to one another [9, 11, 14, 20]. This purportedly extends to ratite birds [42–44]; however, further analysis presented in this study challenges this notion. That is, we identified 26 interchromosomal rearrangements in ostrich compared to the ancestor. Moreover, the question of whether the conserved interchromosomal synteny seen in the macrochromosomes applies to the microchromosomes has hitherto been beyond the resolution of contemporary methodology. This study is the first to classify inter-microchromosomal rearrangements in any species; we provide evidence that interchromosomal rearrangements are nonetheless rare, except in cases (around 1/3 of species) where we already knew that karyotypes were highly rearranged [9]. Ostrich is the exception and it will be interesting to note whether this applies to other ratite birds.

Microchromosomes are not a uniquely avian feature. They are also found in some primitive amphibians (Cryptobranchidae and Hynobiidae have 14–19 pairs [45, 46]), most (but not all) reptiles (snakes have around 20 pairs [47]), but paradoxically not Crocodylia [48] – the closest phylogenetic lineage to birds. Indeed microchromosomes are typical of most amniotes (mammals and crocodilians being exceptions); however, the greatest number and smallest size of microchromosomes are typically found among birds. Burt [49] in a “fission-fusion” hypothesis suggested that most microchromosomes were already present in the common dinosaur ancestor that gave rise to birds (which probably had already evolved a small genome size and karyotype of around 2*n* = 60 including 20 pairs of microchromosomes) but that chromosome fission created the remainder, presumably including the smallest ones. In the current study, the similar number of chromosomes amongst most species but relatively large number of rearrangements between ostrich and all the other birds studied suggest that a basic pattern of 2*n* = 80 (~30 pairs of microchromosomes) became fixed before the Palaeognathae-Neognathae divergence 100 MYA but that interchromosomal rearrangement was still relatively common in birds at the time. Another alternative is that ratite birds underwent further adaptive changes that may be associated with the very different phenotypes present in this clade alone. The paucity of inter-microchromosomal rearrangements between most Neognathae (if the evidence presented here is representative, this would presumably include the 2/3 of Neognathae species where 2*n* = ~80) supports our hypothesis that the microchromosomes represent blocks of conserved synteny at an interchromosomal level. An absence of interchromosomal rearrangement could either suggest an evolutionary advantage to retaining this particular configuration or a lack of opportunity for chromosome rearrangement. The latter might be explained by few recombination hotspots, transposable elements or endogenous retroviruses, all of which have been associated with chromosomal change. Both inter- and intrachromosomal change can arise via these mechanisms, and thus the rapid amount of intrachromosomal but not interchromosomal change in our representative passeriform species, the zebra finch, suggest that there may be an evolutionary advantage to keeping microchromosomes numerous, gene dense, compact and evolutionarily static. Stasis in evolution can, however, arise via alternative interpretations; it may be that the mutational mechanisms underlying chromosomal changes are different in birds or that lack of adaptive value, rather than purifying selection, slows down the rate of chromosomal changes. At the time of writing no sequences have yet been associated with the very smallest of the avian microchromosomes (29–38) and this is an issue that will require rectifying in future avian genome projects using more sophisticated technologies.

The rate of chromosomal change in any eukaryotic organism, and the speciation that ultimately arises from it, is dependent on two factors: the rate of mutation and the rate of fixation [18]. The mutation rate of chromosomes is, in turn, related to the frequency of homologous sites [49]. Repeat structures in general, and transposable elements in particular, provide substrates for chromosomal rearrangement. In a genome that is constrained by size (perhaps, as has been suggested, because of the energy requirements associated with flight [50, 51]), the opportunity for mutation is reduced and only fission (or intrachromosomal rearrangement such as inversion) can occur. This would explain first why the avian genome is the most fragmented of any vertebrate genome (i.e. birds have the most chromosomes) and second why there have been few interchromosomal rearrangements in most species. There are also possible advantages of multiple chromosomes in a karyotype in terms of generating variation, the driver of natural selection. That is, more chromosomes lead to more combinations of gametes as well as an increase in recombination rate as there has to be at least one obligatory chiasma per chromosome. The absence of positive selection for much change in chromosome number is a possible explanation of why there was little fixation of any interchromosomal changes among birds although inbreeding and genetic drift may play a role [18, 49, 52, 53]. Burt [49] suggested that a higher recombination rate is another constraint that has resulted in the properties we most associate with microchromosomes (e.g., high GC-content, low repeats, high gene-density) and led to the maintenance of the typical avian karyotype with both macro- and microchromosomes and few rearrangements between them.

A constraint of overall karyotype structure does not preclude intrachromosomal rearrangements. Indeed there is a correlation between the rates of speciation and intrachromosomal rearrangement [4]. In the current study, the rapid rate of intrachromosomal rearrangement in the zebra finch would argue for a relationship between intrachromosomal rearrangement and speciation in birds given the Passeriformes represent over half of all species. Such mechanisms could be mediated through an increase in localized repeat content. Hotspots of recombination have previously been reported to also play a role [14] and in this study we tested the hypothesis further utilizing “zebra finch only” and “chicken only” breakpoints comparing them to previously reported genetic maps of each species [37, 54, 55]. In chicken, recombination rates were near identical in regions with breakpoints compared to those without. In zebra finch, the difference in rates between regions containing EBRs and regions without EBRs, although similar in magnitude to that previously reported [14], failed to reach statistical significance (at *P* < 0.05). This therefore casts doubt on our original findings, thereby either suggesting that our hypothesis should be rejected or that the numbers in the study were not sufficiently large to reach statistical significance. A further alternative explanation is that the available recombination maps have too low marker density (typically Mb scale) to pick up local recombination rate variation at a sufficiently detailed scale (Kb scale) to detect associations with EBRs. Study of a greater number of species in this manner using high-density linkage maps or population based recombination rate estimates may resolve the paradoxical difference between [14] and the current study.

Some avian species undergo a radical departure from the typical (2*n* = ~80) avian genome organization. The presence of an unusually high chromosome number in the Adélie penguin (2*n* = 96) and a lower than average number in the emperor penguin (2*n* = 72) (but both associated with high degrees of inter-microchromosomal rearrangement) suggest that similar mechanisms can act to either reduce or increase chromosome number rapidly. Evidence from the penguins and the rearranged karyotypes of the Falconiformes and the Psittaciformes suggest that these changes can happen in a relatively short time. Mammals, reptiles and amphibians with larger, repeat-rich genomes have the potential to undergo rapid intra- and interchromosomal rearrangements and the results presented here suggest that birds too can undergo similar changes in certain groups. We are not, however, aware of any evidence to suggest that highly rearranged avian genomes are especially large, or significantly more repeat-rich than other avian genomes. Comparisons of the zebra finch and the budgerigar suggest that mutation rates of chromosomes may well be similarly high in both groups but that they are features associated with exploiting evolutionary niches in certain groups that serve to fix interchromosomal rearrangements, while in others such fixation is prevented and the overall avian karyotype maintained. Such processes are, to date, undiscovered but possible clues might lie in the study of GO terms present in EBRs. In an associated study, a correlation between EBRs and specific avian adaptive features in individual species has been demonstrated. This included forebrain development in budgerigar, one of the six species focused upon in this study and consistent with this species being not only vocal-learner but having distinctive neuronal connections compared to other vocal-learners [37]. As more genomes become available with better assemblies, these analyses may well point to adaptive phenotypic features of individual orders and families.

Finally, we observed that it appears to be the chicken that seems to have undergone the fewest chromosomal changes compared to the ancestor. There are interesting parallels between this study and another study [56] examining sex chromosome evolution. While our data demonstrates that autosomes have been reorganized least in chicken chromosomes 1–5 in comparison to the common avian ancestor, Zhou *et al.*[56] conclude that the ancestral sex chromosome organization is observed closer to that of the Palaeognathae (ostrich and emu). Zhou *et al.*[56] show less degradation of the sex chromosomes and a closer synteny to the lizard. As, in this study, we only examined the Z chromosome in the Neognathae (for the reasons given), further studies will be required to establish whether sex chromosomes and autosomes preserve their ancestry differently in the different lineages. The question also arises of whether chicken and related species, having undergone the fewest chromosomal changes, have undergone the fewest adaptive changes compared to the avian ancestor. Most authors agree that the dinosaur ancestors of birds were bipedal and terrestrial, relatively small (small size being an immediate pre-adaptation to flight) and had limited flying ability, not unlike Galliformes [57]. On the other hand, the earliest known Ornithurae along the presumed direct line to modern birds were either fully aquatic or amphibious (e.g., *Gansus*[58]) and details of their anatomy, including webbed feet, have been likened to ducks [59, 60]. The oldest relatively certain fossil representative of Neornithes (modern birds) is aquatic, and identified as a Galloanseres (e.g., *Vegavis*[61]). However, the fossil record may be difficult to interpret due to geographic and depositional sampling biases, limited understanding of functional anatomy, and the uncertainty that avian ancestors were ecologically and behaviorally typical of the larger groups to which they belonged. As an independent record of the actual substance of inheritance of living birds, genomic characteristics such as chromosomal arrangement complement a fossil record that may imperfectly represent actual neornithine forebears. Thus, chromosomal rearrangements may provide information on the ecological adaptations of avian ancestors that the fossil record may never be able to establish unambiguously [62].

## Conclusions

In summary, this study represents the most comprehensive appraisal of changes in overall avian genome structure hitherto reported. We provide further insight on previously reported roles of genetic recombination in chromosome rearrangement and on the functional significance of karyotype stability in the avian genome. Here, we establish that the chicken lineage contains the fewest number of chromosomal changes compared to the dinosaur ancestor relative to the other five species studied. At this stage it would be unwise automatically to infer that this means that the chicken has the fewest number of adaptive changes also. This will nonetheless be the topic of future study.

## Methods

### Presentation of multiple avian genome assemblies

In order to present and visualize comparative cytogenetics and identify HSBs and EBRs in multiple avian species, an interactive, comparative chromosome browser Evolution Highway was used [38]. All blocks of synteny were identified and displayed relative to chromosomes of the reference chicken genome (ICGSC Gallus_gallus-4.0/galGal4). Evolution Highway was used to display the sequence coordinates of all syntenic fragments (SF) and HSBs in each genome [37]). We made use of the set of HSBs and SFs that contained rearrangements that are ≥ 300 Kb in the reference genome. This set, together with two other separate sets that visualize HSBs and SFs that are larger than 100 Kb and 500 Kb in the reference genome, is publicly available from the Evolution Highway website [36] (Figure 1) and are further described in [37].

For the purposes of this study, 21 avian genomes plus one outgroup species were utilized to address the questions set out in the Background section and made up of the following: of these 21, 17 were recently sequenced and presented [2] including common cuckoo, peregrine falcon, American crow, little egret, crested ibis, domestic pigeon, hoatzin, golden-collared manakin, medium ground finch, downy woodpecker, Adélie penguin, emperor penguin, Anna’s hummingbird, chimney swift, killdeer, budgerigar and ostrich. Conserved blocks of synteny are presented as scaffolds (scaffold 1 being the largest and the rest numbered accordingly to size) in relation to chicken chromosomes. Chromosome-level assembly and analysis of conserved synteny had been previously reported for the largest (macro-) chromosomes of chicken, turkey and zebra finch [14, 20, 21]. Thus, the turkey (TGC Turkey_2.01/melGal1) and zebra finch (WUGSC 3.2.4/taeGut1) genomes were presented in Evolution Highway with reference to published chromosome number (e.g., chromosome 11 in chicken corresponds to chromosome 12 in duck and 13 in turkey; see Figure 1). Chromosome-level assembly of the Pekin duck genome was constructed from available genome scaffolds [63] using an original RH mapping approach through hybrid sequencing (Faraut *et al.*, personal communication). Pekin duck was added and presented with reference to published chromosome number. The Carolina anole was the only reptile outgroup genome available with reference to whole chromosomes and therefore this was chosen for this study as the outgroup for reconstruction of the ancestral chromosomes (see the sub-section *Establishment of ancestral avian karyotypes*).

Of the 17 newly sequenced species, two (ostrich and budgerigar) were selected for studies involving reconstruction of the ancestral chromosomes. These species, thanks to optical mapping, had the largest N50 (>10 Mb) and were also the species on which we performed zoo-FISH studies due to the availability of material for chromosome preparation. These and the remaining 15 species were used for defining EBRs to compare with recombination rate and for establishing interchromosomal conserved synteny among the microchromosomes [37].

### Karyotype and zoo-FISH analysis

For chromosome analysis, rapidly dividing embryonic fibroblasts or white blood cells were arrested in metaphase using colchicine (Sigma), swollen using 75 mM KCl and fixed to glass slides using 3:1 methanol : acetic acid mix. Metaphases were stained with a combination of DAPI and propidium iodide in VECTASHIELD® antifade medium (Vector Laboratories). Image capture involved an Olympus BX61 epifluorescence microscope with cooled CCD camera; SmartCapture system and SmartType software (Digital Scientific UK) were used for capturing and karyotyping purposes, respectively. Microchromosome paints described elsewhere [21] were generated by flow cytometry, then amplified and directly labeled with FITC using DOP-PCR. BAC clone DNAs were used to verify chromosome paint alignment and were extracted by miniprep (QIAprep Spin Miniprep Kit, QIAGEN), then directly labeled by nick translation with FITC or Cy3.5.

For FISH, metaphases were probed with chicken chromosome paints and BACs generated above. Briefly, probes were dissolved in a formamide buffer and applied, under a coverslip, and then sealed using rubber cement. Simultaneous denaturation of probe and genomic DNA on a 75°C hotplate preceded hybridization at 37°C (overnight for same species FISH, three days for zoo-FISH). Post-hybridization washes (2 minutes in 0.4 × SSC at 73°C; 30 seconds in 2 × SSC/0.5% Tween 20 at room temperature) were followed by chromosome counterstaining using VECTASHIELD® anti-fade medium with DAPI and viewed as above using epifluorescence and SmartCapture (Digital Scientific UK).

### Establishment of ancestral avian karyotypes

In total six avian species (chicken, turkey, duck, zebra finch, ostrich and budgerigar) plus one lizard outgroup species (Carolina anole) were chosen for reconstruction of the ancestral karyotypes (for the reasons given in the sub-section *Presentation of multiple avian genome assemblies*). A combination of bioinformatics, zoo-FISH and karyotyping allowed us to make reconstructions of the order and orientation of scaffolds and thence the ancestral chromosomes. To reconstruct a putative avian ancestor as inferred from orthology maps the Multiple Genomes Rearrangements and Ancestors (MGRA) tool on the Algorithmic Biology Lab web server at St. Petersburg Academic University of the Russian Academy of Sciences [64, 65] was used as follows: using Evolution Highway, pairwise alignments for turkey, duck, zebra finch, budgerigar and ostrich were visualized relative to the chicken whole genome sequence as a reference at the 300 Kb resolution. The orthology map of the Carolina anole, also visualized by Evolution Highway, was used as an input for the MGRA program and included in the analysis as an outgroup. Orthologous regions observed in all the species compared were defined as msHSBs and served as MGRA inputs for individual genomes. The hypothetical ancestral genome was determined using the phylogenetic tree information for this set of six species [35].

For chromosomes 1–5, 80% of the avian genomes were also represented by orthologous sequences in the Carolina anole outgroup. In this case we could therefore reconstruct the ancestral chromosomes for all birds. For chromosomes 6–28 and Z, we used ostrich as the outgroup (thus only drawing conclusions about the Neognathae), as only ~9% of the genome had orthologous sequences represented in the lizard outgroup. Where the ostrich and Neognathae ancestor had the same arrangement of HSBs, we could infer the avian ancestor (as with chromosome 11, Figure 3).

In order to test the robustness of our analysis in a series of additional MGRA simulations and iterations, we established if exclusion of one species at a time from the set of six species would affect the overall pattern of the reconstructed avian ancestor genome organization.

### Reconstruction of evolutionary events guided by MGRA

The positions of CARs and HSBs or SFs within each species genome were noted, allowing correlation with our previously published FISH based physical mapping data in chicken turkey, duck and zebra finch [12–14] and that derived by cross-species chromosome painting in former publications [66, 67] and in the current study. These data were previously acquired by cross-species FISH of chicken BACs and chromosome paints onto turkey, duck, ostrich and budgerigar chromosomes, and same-species FISH of orthologous zebra finch BACs onto zebra finch chromosomes.

The available karyotypic, FISH and bioinformatic data were combined to generate the “best-fit” model for chromosomal evolution in the six avian species of interest, i.e. the one with the minimum number of rearrangements. The MGRA tool was used on the whole genome datasets to reconstruct the evolutionary events that, most parsimoniously, led to the arrangement seen in the extant species. For the most part, the changes suggested by MGRA were accepted as the most parsimonious involving the minimum inversions for intrachromosomal rearrangements and fissions/fusions for interchromosomal rearrangements (the process of defining the inversions is illustrated in Figure 3; see also [20]). In cases where apparent interchromosomal rearrangements (such as translocations) had occurred, the MGRA solution was cross-referenced with the reconstructions on a chromosome-by-chromosome basis using the Multiple Genome Rearrangements (MGR) tool [68, 69] and with zoo-FISH data. In cases of disagreement on the pattern of rearrangements, three independent observers with extensive cytogenetic expertise manually checked and decided the rearrangement pattern. When a whole, otherwise independent, block (scaffold or chromosome) was classed as inverted, this was counted in the analysis as a true inversion if a different orientation was recovered for two or more species (example shown in Figure 3b for chromosome 11 in zebra finch).

### Identification of EBRs and breakpoint reuse

We used the EBRs defined in [37] that involved a single reference chromosome (intrachromosomal EBRs) and more than one reference chromosome (interchromosomal EBRs) in target species’ chromosomes or scaffolds [70]. Interchromosomal EBRs delineated interchromosomal rearrangements, which were then compared with published chromosome number [9], or more specifically deviation from *n* = 40; correlation coefficient *R*^*2*^ was calculated using Microsoft Excel. In order to determine breakpoint reuse, the series of possible rearrangements from the common avian ancestor (with lizard as the outgroup, chromosomes 1–5) or Neognathae ancestor (with ostrich as the outgroup, chromosomes 4p, Z and 6–28) to each species was considered, and for each rearrangement, the segment ends flanking the breakpoints were noted. Within each lineage, the number of times a segment end was involved in a rearrangement was counted and reuse classified if it occurred more than once in any lineage or between lineages.

### Recombination rate analyses

We used the chicken- and finch-specific EBRs defined in [37] to compare with chicken-specific recombination rates and zebra finch-specific EBRs with zebra-finch recombination rates. This differed from our previous approach [14] in which we examined all EBRs between three species compared to the zebra finch genetic map. Zebra finch-specific EBRs coordinates initially identified in chicken chromosomes were translated into zebra finch chromosome coordinates (WUGSC 3.2.4/taeGut1) using the correspondence between coordinates of finch HSB boundaries in the chicken and finch chromosome assemblies [37]. In this way all chicken-specific and zebra finch-specific EBRs identified at 300 Kb resolution were compared directly with genetic maps in chicken and zebra finch, respectively.

We obtained sex-averaged recombination rate estimates for 1 Mb non-overlapping windows by comparing genetic and physical positions of SNPs distributed along the chicken and zebra finch genomes (data from [54, 55]). To assess if the recombination rate differed between regions with and without chromosomal breakpoints, we partitioned the recombination data into two classes, one with windows containing at least one breakpoint and one with windows without breakpoints, using the zebra finch and chicken breakpoint data [37]. We applied a non-parametric test (Wilcoxon’s rank sum test with continuity correction as implemented in R [71]) to assess the level of significance for the difference in recombination rates between classes. Since the sample size differed considerably between classes (i.e. windows not containing EBRs vastly exceeded those that contained EBRs) we also applied a randomization test in R [71]. We randomly sampled the same number of windows as those containing EBRs in each respective taxon (*n* = 31 for zebra finch, *n* = 35 for chicken) from the entire sample 10,000 times. Lastly, we calculated the average recombination rate in the random sample of windows for each iteration to obtain an expected distribution.

### GO analysis of microchromosomes

In order to ask whether individual microchromosomes were enriched for specific GO categories, whole gene sets for each microchromosome were collated and loaded both into DAVID [72, 73] and GOEAST [74, 75]. Specifically, Ensembl gene ID data and gene name for each microchromosome were extracted from the BioMart Ensembl Genes 75 Database [76, 77], using galGal4 as the dataset. In order to eliminate any “significant” results arising through the presence of multiple copies of genes in the same family being present on the same chromosome, gene families were reduced to a single representative member. Downloaded gene IDs and gene names were then copied into a spreadsheet for further analysis using DAVID and GOEAST. Gene IDs for each microchromosome were uploaded into DAVID Bioinformatics Resources 6.7, using Ensembl Gene ID as the list identifier and subsequently analyzed using the Functional Annotation Clustering tool. Cluster data from each microchromosome gene list output was downloaded into Microsoft Excel and filtered using an enrichment score of 1.3 and above and a *P* value less than 0.05 to edit the list for clusters considered to be significant. BioMart (Ensembl) derived gene names for each microchromosome were also uploaded into GOEAST using *Gallus gallus* as the reference. Batch-gene analysis was performed by GOEAST, and enriched GO term outputs with a *P* value less than 0.05 were considered to be significant. The GO results obtained from GOEAST were downloaded into Microsoft Excel and presented with graphic files created directly from GOEAST for each microchromosome where results were available. Finally, in order to correct for multiple sampling error, an FDR threshold of 0.05 was used.

## Authors’ information

Michael N Romanov and Marta Farré, joint first authors.

Denis M Larkin and Darren K Griffin, joint last and corresponding authors.

## Electronic supplementary material

Additional file 1: Table S1: BAC clones used to confirm chromosome paint assignments. (DOCX 73 KB)

## Abbreviations

BAC: Bacterial artificial chromosome
CAR: Contiguous ancestral region
cM: Centimorgan
CNV: Copy number variation
EBR: Evolutionary breakpoint region
FDR: False discovery rate
FISH: Fluorescent in situ hybridization
GC: Guanine-cytosine
GO: Gene ontology
HSB: Homologous synteny block
Kb: Kilobase
Mb: Megabase
msHSB: Multispecies homologous synteny block
MGR: Multiple Genome Rearrangements
MGRA: Multiple Genomes Rearrangements and Ancestors
MY: Million years
MYA: Million years ago
SD: Standard deviation
SF: Syntenic fragment
SNP: Single nucleotide polymorphism.
