# Supplementary material for: Reconstruction of gross avian genome structure, organization and evolution suggests that the chicken lineage most closely resembles the dinosaur avian ancestor
Source: BMC Genomics. 2014 Dec 11;15(1):1060. doi: 10.1186/1471-2164-15-1060 (PMC4362836; doi:10.1186/1471-2164-15-1060)
Supplement: Supplementary file 1 — Additional file 1: Table S1: BAC clones used to confirm chromosome paint assignments. (DOCX 73 KB) [file 12864_2014_6932_MOESM1_ESM.docx]

| **Chromosome** | **BAC** | | | |
| --- | --- | --- | --- | --- |
| **Number** | **Start Position** | **End Position** | **Clone Name** | **Span** |
| 10 | 218,904 | 410,857 | CH261-179N4 | 191,954 |
| 10 | 22,346,215 | 22,540,226 | CH261-112C21 | 194,012 |
| 11 | 2,031 | 196,377 | CH261-108G7 | 194,347 |
| 11 | 21,599,599 | 21,797,804 | CH261-26N2 | 198,206 |
| 12 | 9,046 | 174,640 | CH261-36I16 | 165,595 |
| 12 | 20,282,384 | 20,468,673 | CH261-88O8 | 186,290 |
| 13 | 513,090 | 698,911 | CH261-154L1 | 185,822 |
| 13 | 18,503,120 | 18,659,934 | CH261-29I18 | 156,815 |
| 14 | 75,061 | 264,260 | CH261-104G13 | 189,200 |
| 14 | 15,620,338 | 15,819,411 | CH261-23C6 | 199,074 |
| 15 | 37,719 | 230,572 | CH261-131E4 | 192,854 |
| 15 | 12,766,676 | 12,953,903 | CH261-40D6 | 187,228 |
| 16 | 7,438 | 178,006 | CH261-177D20 | 170,569 |
| 17 | 531,124 | 691,083 | CH261-18C4 | 159,960 |
| 17 | 10,978,652 | 11,167,736 | CH261-154L3 | 189,085 |
| 18 | 159,671 | 345,409 | CH261-67N15 | 185,739 |
| 18 | 10,715,138 | 10,889,646 | CH261-72B18 | 174,509 |

Supplementary table 1. BAC clones used to confirm chromosome paint assignments
